# Light people: Professor Ioannis Papakonstantinou

**DOI:** 10.1038/s41377-026-02531-y

**Published:** 2026-11-04

**Authors:** Zhijie Xu, Siqiu Guo

**Affiliations:** 1 Photonic Innovations Lab, Department of Electronic and Electrical Engineering, University College London, London, UK; 2 Light Publishing Group, Changchun Institute of Optics, Fine Mechanics and Physics, Chinese Academy of Sciences, Changchun, China; 3 Ji Hua Laboratory, Foshan, 528200 China

**Keywords:** Optoelectronic devices and components, Solar energy and photovoltaic technology

## Abstract

From optical communications and nanophotonics to sustainable cooling and climate-resilient agriculture, Professor Ioannis Papakonstantinou has built a research career around a central question: how can light be controlled and engineered to address real-world challenges? As Professor of Photonics and Nanofabrication at University College London and Head of the Photonic Innovations Lab, his work spans fundamental light–matter interactions, nanostructured and multifunctional surfaces, luminescent solar concentrators, optical communications and passive thermal management. Across these diverse areas, his research combines theoretical modelling, nanofabrication and experimental validation, while maintaining a strong emphasis on translating photonic concepts into scalable technologies (Figs. 1, 2, 3, 4).

In this issue of Light People, we are delighted to feature Professor Papakonstantinou and explore the scientific ideas and personal experiences that have shaped his interdisciplinary career. From reimagining luminescent solar concentrators for optical communications and horticulture to developing adaptive materials and passive radiative-cooling technologies, the conversation reveals a research philosophy grounded in fundamental physics but driven by applications in sustainability and society. We also discuss his views on scientific risk, collaboration, research culture, and the role photonics may play in addressing some of the major technological and environmental challenges of the coming decades.

**Figure:**
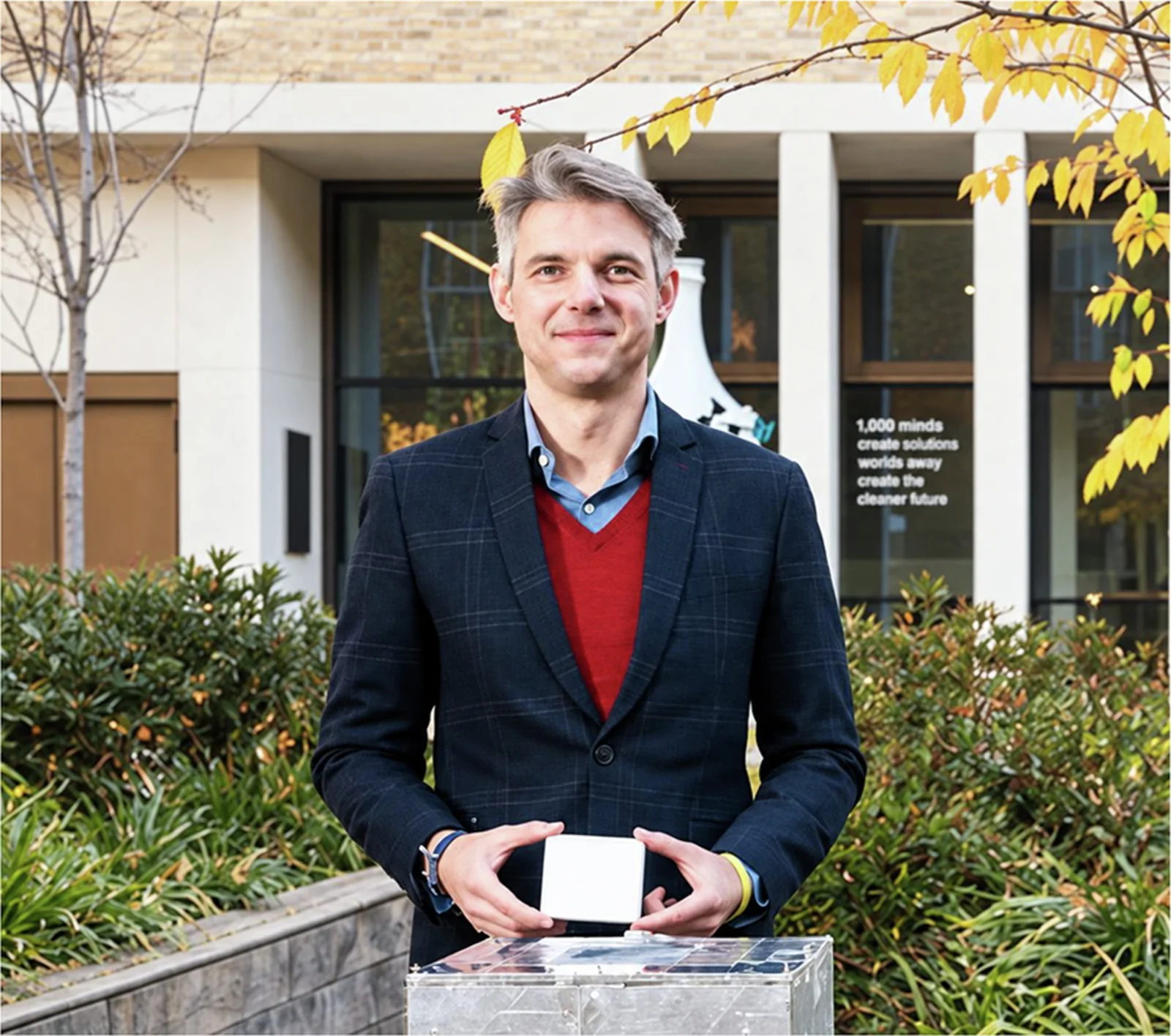

Ioannis Papakonstantinou is Professor of Photonics and Nanofabrication in the Department of Electronic and Electrical Engineering at UCL and leads the Photonic Innovations Lab. Before joining UCL, he worked at CERN (European Organization for Nuclear Research) and Sharp Laboratories of Europe. His research focuses on light–matter interactions at the micro- and nanoscale, with applications spanning mid-infrared photonics, radiative cooling, luminescent solar concentrators, biomedical devices, climate resilient-agriculture and more. He has been the recipient of multiple awards including four grants from the European Research Council and two Marie-Curie fellowships. Ioannis also holds a diverse portfolio of 14 patent families in areas including optoelectronic systems, liquid crystal displays, photonic security features, biomedical devices, and radiative cooling technologies.

**Q1. Your research spans nanophotonics, nanofabrication, luminescent solar concentrators, optical communications, smart surfaces and radiative cooling. What is the common scientific thread that connects these diverse research directions?**

The thread is light itself. Every one of these directions is about controlling how light is generated, scattered, guided, absorbed or emitted. What changes from one project to the next is the wavelength and the application, not the fundamental physics. Electromagnetism, the mathematics of wave scattering and propagation or the way light interacts with matter are the same whether we are designing a nanostructured surface, a luminescent waveguide or a radiative cooler. Once you are fluent in these fundamentals, moving between applications is far less of a leap than it appears from the outside.

There is a second, more personal thread. I am drawn to problems where photonics can make a meaningful difference to how we live, whether that is exchanging information, growing food or improving the energy efficiency of our homes and buildings. That ambition is what has pulled the group from one direction to the next.

**Q2. In 2021, you published a fascinating paper in Light: Science & Applications entitled “Bandwidth Limits of Luminescent Solar Concentrators as Detectors in Free-Space Optical Communication Systems.” Although LSCs were originally developed for solar-energy harvesting, over the past decade they have also emerged as wide-field-of-view receivers for optical communications. What first drew you to this less conventional application of LSCs, and how do you see this research area developing in the future?**

The idea was first proposed by a group at Oxford, and it landed at exactly the right moment for me. I had come to the view that LSCs were approaching their limits as electricity generating devices and that the next wave of innovation would have to come from a different direction. My PhD was in optical communications, so the notion of using an LSC as a wide-field-of-view, high-speed receiver resonated immediately (Fig. 1).

**Fig. 1:**
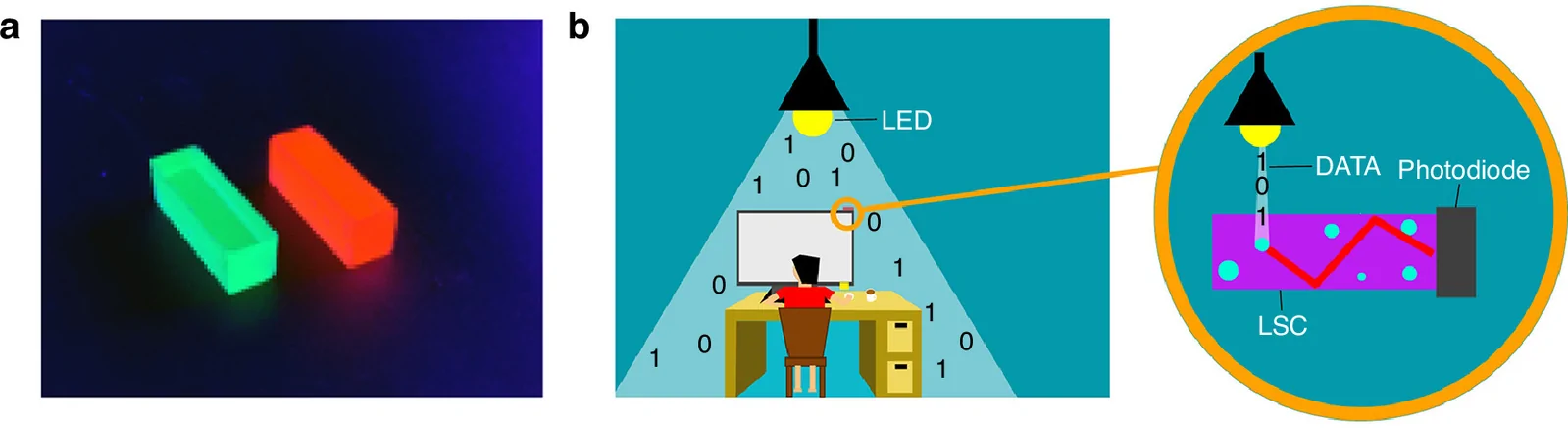
**a** Photograph of Lumogen Red 305- and CsPbBr3-doped LSCs (0.67 × 0.67 × 2 cm) under illumination by an ultraviolet lamp. **b** Graphic depicting the principle of operation of an LSC-VLC system^1^

What I also saw were two clear gaps. Nobody seemed to have established the information density an LSC could actually carry, and there were no tools to model and optimise these devices as receivers rather than as energy harvesters. The Light: Science & Applications paper set out to close both gaps by adding a new dimension to existing LSC modelling tools: the time domain. Looking ahead, I expect LSC receivers to find their place in short-range, high-bandwidth links, indoor optical wireless (Li-Fi) and other free-space communication systems. I am also very keen to explore the integration of LSCs with sensing systems. The challenge now is to engineer luminophores that combine short emission lifetimes, which set the bandwidth, with high quantum yield, which sets the signal-to-noise ratio, and to optimise the internal lightguide geometry around them.

**Q3. In addition to their use in optical communications, your recent research has explored the application of LSCs in horticulture. What motivated you to pursue this direction, and how do horticultural LSCs differ fundamentally from conventional LSCs in terms of their design objectives and operation?**

The motivation is personal. I grew up in Trikala, a small town in central Greece, where many of my uncles and aunts were farmers, and I spent much of my school holidays there. That left me with a lasting respect for farming and a clear sense of how hard it is. Combine that with my concern about climate change and the challenge of feeding a growing population, shifting part of the group’s effort towards agriculture was a natural step.

The design objectives are almost the inverse of a conventional LSC. A conventional LSC is built to capture as many photons as possible and guide them to a photovoltaic cell at the edge with light escaping through the faces being considered as loss. A horticultural LSC is built to do the opposite. It should transmit as much useful light as possible to the crop canopy beneath, while converting only the photons plants use poorly, mostly in the UV and green, into wavelengths that drive photosynthesis most efficiently (Fig. 2). The figure of merit is no longer electrical output but crop yield and light-use efficiency, and the intersection of light and biology is a hardly understood area that fascinates me deeply.

**Fig. 2:**
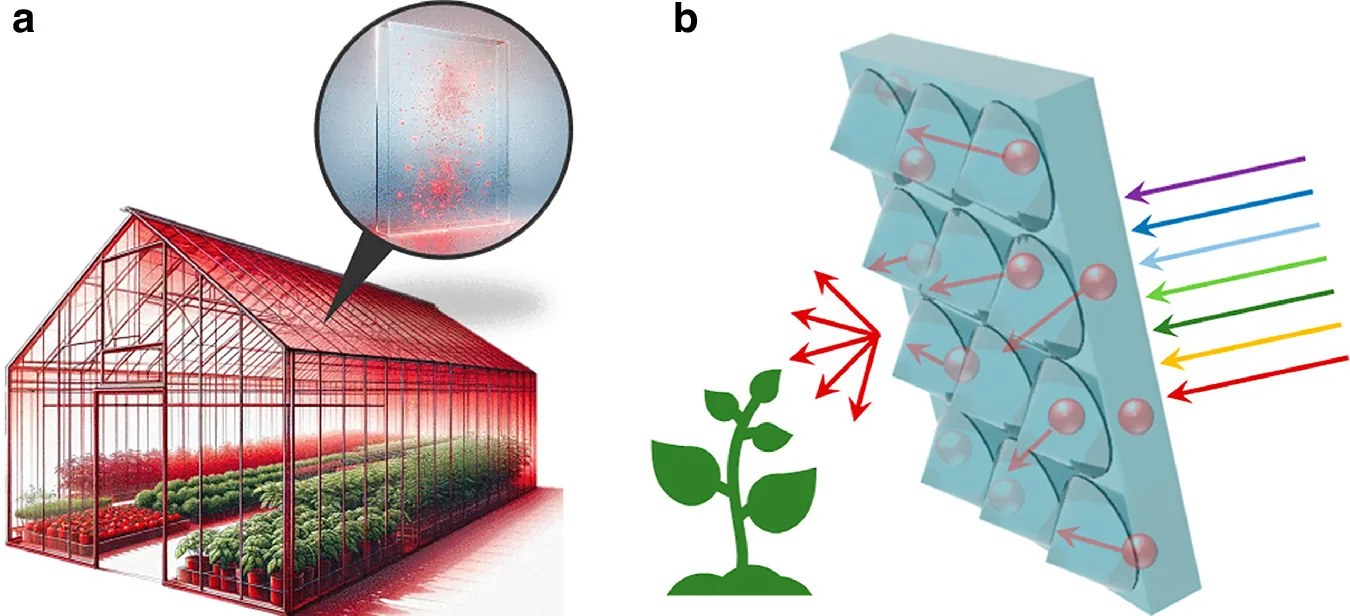
Luminescent solar concentrators for horticultural applications. **a** Concept of a greenhouse incorporating horticultural luminescent solar concentrators (HLSCs) to spectrally tailor sunlight for plant growth^2^. **b** Schematic illustration of a microstructured HLSC, in which incoming sunlight is absorbed and re-emitted as red light, while surface microstructures help extract the converted light towards the plants^3^

**Q4. Your recent research has increasingly focused on passive radiative cooling, thermochromic materials and multifunctional optical surfaces. Congratulations on receiving an ERC Advanced Grant for this research programme. As Europe experiences increasingly intense and frequent summer heatwaves, could you explain the programme’s central idea and how it might lead to practical, low-energy cooling solutions?**

Thank you. Like the horticulture work, radiative cooling has roots in my upbringing. Summers in Greece meant heatwaves, and without air conditioning we spent long stretches in my grandparents’ basement because it was the coolest part of the house. Climate change is making our dependence on air conditioning less and less sustainable, so finding alternatives is a priority.

The central idea behind my ERC grant starts from a striking gap. Thermodynamics tells us that radiative cooling can reach temperatures well below freezing, yet almost every system demonstrated so far achieves only a few degrees below ambient. That gap reminded me of the early days of solar cells, which operated at a small fraction of their theoretical limit, and it convinced me there was room for a step change. The only demonstration of sub-zero temperatures at the time, from Shanhui Fan’s group at Stanford, relied on vacuum pumps and cryogenic chambers to suppress conduction and convection. This was an excellent proof of concept, but not something you could put on a roof.

The programme sets out to achieve the same effect fully passively, using materials and fabrication methods that are scalable and potentially low-cost (Fig. 3). We are at the beginning of that journey, but if we succeed the technology could reduce the need for air conditioning, cut water consumption in cooling towers, and be integrated into food cold chains, displacing active cooling wherever it is feasible to do so.

**Fig. 3:**
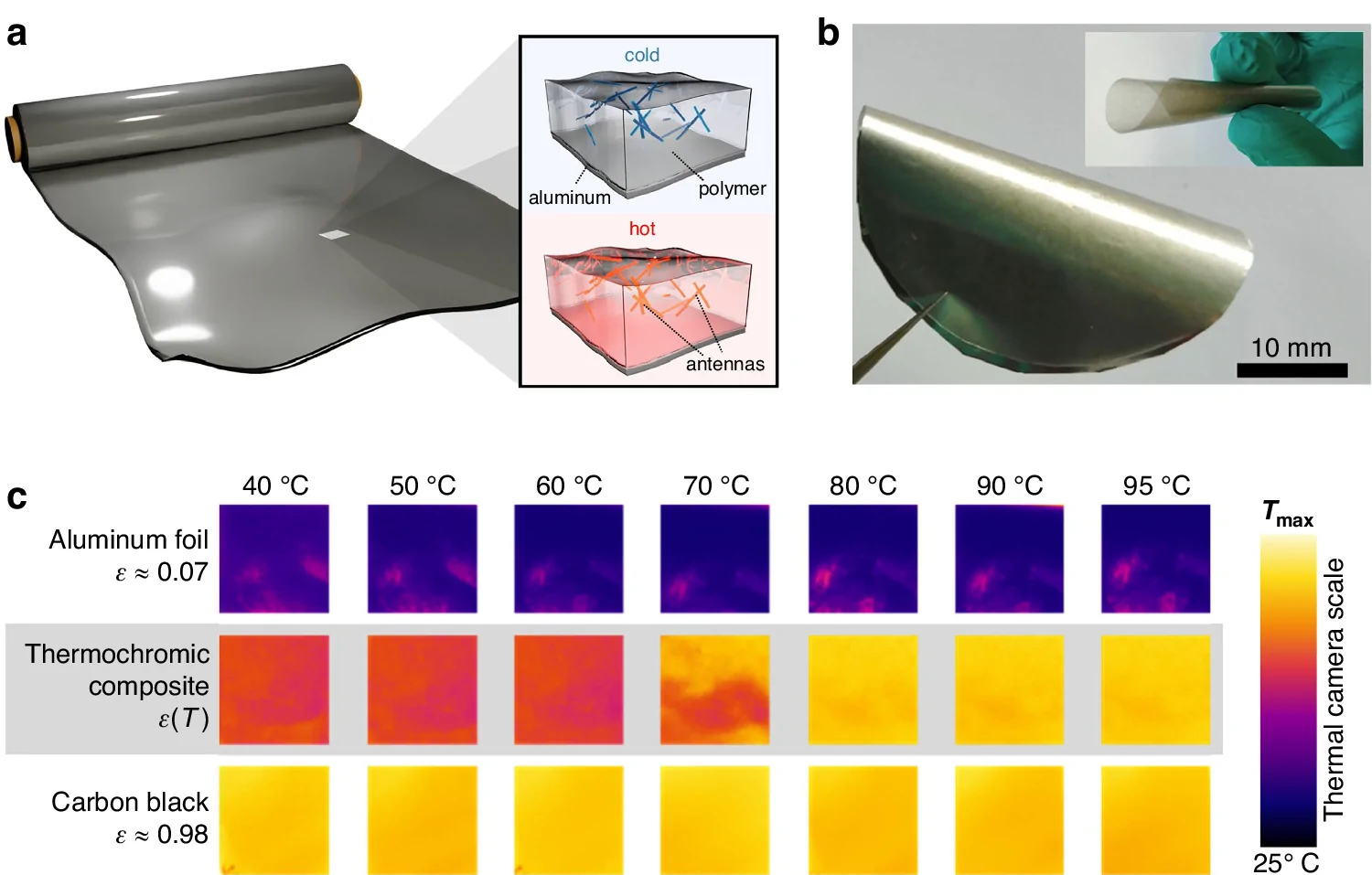
Flexible thermochromic material for passive temperature regulation. **a** Working principle of the composite, which changes its thermal emission as temperature increases. **b** Photograph of the flexible film. **c** Thermal images showing how its apparent thermal emission changes with temperature compared with aluminium foil and carbon black^4^

Feasibility matters to me. Too many fields have been overhyped in the past and then burned out, and I would not want to see the same happen to radiative cooling. And while I do not believe in a “silver bullet” solution that will magically solve all our cooling problems, I firmly believe that radiative cooling has a place in that conversation.

**Q5. Looking beyond your own research, where do you believe photonics can make the most meaningful contribution to global sustainability?**

Almost everywhere you look. Optical computing and interconnects can drive down the energy cost per bit at a time when data centres are becoming a significant load on the grid. Solar cells that are cheaper, transparent or flexible can reach surfaces that silicon never will and displace more fossil fuel. Mid-infrared photonics can harvest waste heat and turn it into electricity. Photonic sensors can make industrial processes, agriculture and buildings measurably more efficient. The list is effectively open-ended, and what excites me is that photonics is not a single solution but an enabling layer that underpins nearly every part of the green transition.

**Q6. When you founded the Photonic Innovations Lab at UCL in 2011, what kind of scientific culture did you want to create? How has that original vision evolved as the group has grown?**

I wanted a group built on deep mathematical and physical understanding. I place great emphasis on fundamentals, because researchers who develop deep understanding at that level are more creative and more capable of recognising when something new is possible. They also move across fields and disciplines far more easily, which matters more than ever now that traditional research silos are being dismantled. On top of that, I wanted a group that was curiosity-driven and had both the freedom and the courage to pursue any idea that interested it, provided applications were exclusively for civilian purposes and could make a positive contribution to the world.

That vision has held. What has evolved is the ambition. As the group has grown, we have moved from demonstrating principles to scaling up and tackling real problems in sustainability, food and communications, where the outcome matters beyond the laboratory (Fig. 4). The fundamentals are still the foundation but merely pointing at bigger targets now.

**Fig. 4:**
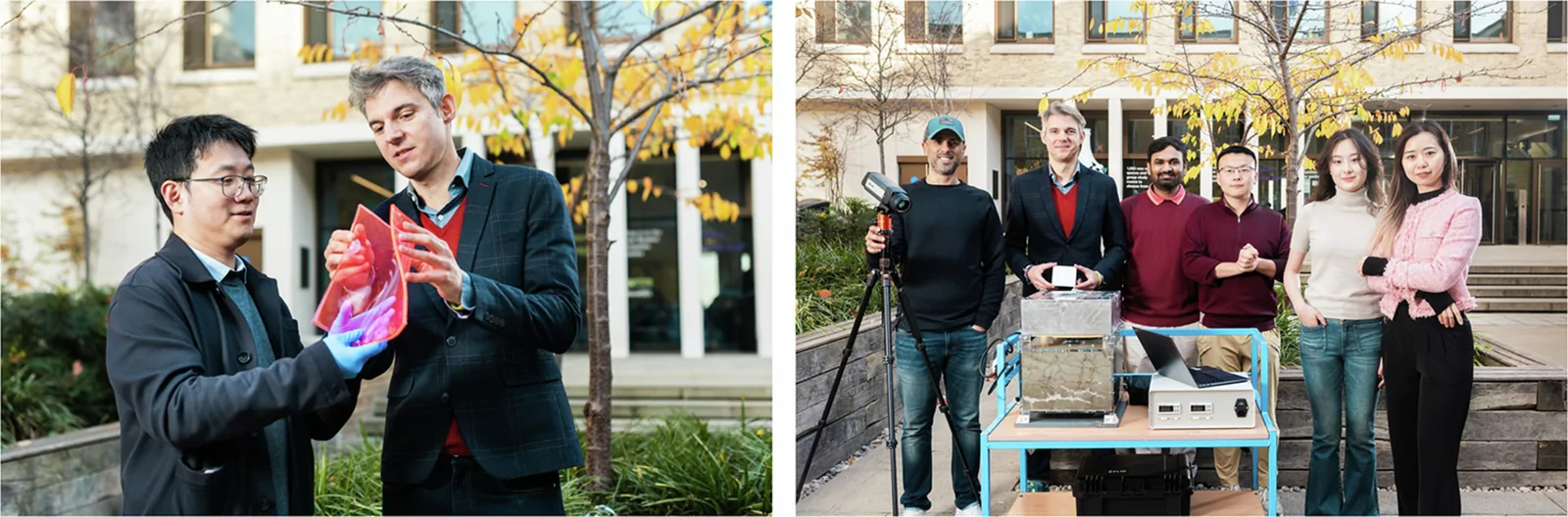
Photonic Innovations Lab, University College London

**Q7. Before returning to UCL, you worked at Sharp Laboratories of Europe, where research was closely connected to technological development. Could you share what that experience taught you about the relationship between fundamental science and practical innovation, and how it has influenced the way you now approach academic research and collaborate with industry?**

I have always been attracted by problems that sit between disciplines. Often, the most interesting questions appear at the boundary between fields: where one community has a practical challenge and another has a method or concept that can help solve it. Metrology naturally encourages this way of working. To measure a complex object, you may need optics, mechanics, computation, statistics, materials knowledge and manufacturing understanding. No single discipline is enough. This is especially true for modern optical metrology, where the instrument, the algorithm and the physical model are deeply connected. Working across fields has made my research more creative and more useful. It has allowed me to bring ideas from physics into engineering, from machine vision into metrology, from quantum estimation into surface measurement, and from manufacturing problems back into fundamental measurement science. It has also influenced how I build collaborations. I like working with people who see the same problem from different angles. This can be challenging, because different communities use different languages and standards of evidence, but it is also where genuinely new ideas often emerge.

**Q8. Over the course of your career, you have explored many different ideas and research directions, and inevitably not every project has unfolded as expected. Could you share one such experience with us? Looking back, how did that setback change the way you evaluate scientific ideas or choose research directions today?**

I tell every new PhD student the same story about the hundreds of ideas that led nowhere. I also keep a list of every rejected grant proposal, which runs to countless pages. I show it to people to make the simple point that success does not mean succeeding every time you try. It comes from persistence and sustained effort, above all when things appear to be going wrong, which is precisely the moment to double down.

Setbacks have changed how I work. I value the limited human and financial resources at our disposal far more than I once did, and I try to deploy them more efficiently. The danger is that this pushes you towards low-risk ideas, so I keep reminding myself of the importance, and the excitement, of high-risk, high-gain research, which is very much the ERC ethos.

**Q9. Outside the laboratory, what activities, places or routines help you disconnect from technical problems? How does time away from research influence your creativity and the way you approach scientific questions?**

Sport, reading and travel. I have tried many sports at one time or another but these days time is limited, so a couple of gym sessions a week and some cycling keep me happy. The ancient Greeks held that a healthy mind comes through a healthy body, and I believe that.

Family comes first. Being there for my children is a major priority, and I make sure I am wherever I am needed. I also have a close group of friends from school days with whom we talk almost daily on a shared WhatsApp group, mostly to make fun of each other. Humour is the best medicine against stress and one of humanity’s finest inventions.

I read widely. My last few books were on philosophy, neuroscience and linguistics, read in parallel, which probably means I barely understood anything. It still astonishes me how much of what was written a long time ago, take Plato for example, remains as relevant today as ever.

All of this shapes how I approach science. I try to come to a problem with an open mind and without preconceptions. Knowing the literature is essential, but the trap is to become confined by it, when real innovation means moving beyond what already exists. I look at a problem holistically, from both the theoretical and the experimental side, and keep trying new approaches until it is resolved.
